# The effect of facility-based antiretroviral therapy programs on outpatient services in Kenya and Uganda

**DOI:** 10.1186/s12913-017-2512-9

**Published:** 2017-08-16

**Authors:** Alexandra Wollum, Emily Dansereau, Nancy Fullman, Jane Achan, Kelsey A. Bannon, Roy Burstein, Ruben O. Conner, Brendan DeCenso, Anne Gasasira, Annie Haakenstad, Michael Hanlon, Gloria Ikilezi, Caroline Kisia, Aubrey J. Levine, Samuel H. Masters, Pamela Njuguna, Emelda A. Okiro, Thomas A. Odeny, D. Allen Roberts, Emmanuela Gakidou, Herbert C. Duber

**Affiliations:** 1 Institute for Health Metrics and Evaluation, University of Washington, 2301 5th Ave., Suite 600, Seattle, WA 98121 USA; 2 Medical Research Council Unit, Banjul, The, Gambia; 3 African Leaders Malaria Alliance, Kampala, Uganda; 4 Harvard School of Public Health, Boston, MA USA; 5 Infectious Diseases Research Collaboration, Mulago Hospital Complex, Kampala, Uganda; 6 Action Africa Help-International, Nairobi, Kenya; 7 University of North Carolina at Chapel Hill, Chapel Hill, NC USA; 8 Afya Resource Associates, Nairobi, Kenya; 9 Bill & Melinda Gates Foundation, Seattle, WA USA

**Keywords:** Antiretroviral therapy, HIV/AIDS, Health systems, Kenya, Uganda

## Abstract

**Background:**

Considerable debate exists concerning the effects of antiretroviral therapy (ART) service scale-up on non-HIV services and overall health system performance in sub-Saharan Africa. In this study, we examined whether ART services affected trends in non-ART outpatient department (OPD) visits in Kenya and Uganda.

**Methods:**

Using a nationally representative sample of health facilities in Kenya and Uganda, we estimated the effect of ART programs on OPD visits from 2007 to 2012. We modeled the annual percent change in non-ART OPD visits using hierarchical mixed-effects linear regressions, controlling for a range of facility characteristics. We used four different constructs of ART services to capture the different ways in which the presence, growth, overall, and relative size of ART programs may affect non-ART OPD services.

**Results:**

Our final sample included 321 health facilities (140 in Kenya and 181 in Uganda). On average, OPD and ART visits increased steadily in Kenya and Uganda between 2007 and 2012. For facilities where ART services were not offered, the average annual increase in OPD visits was 4·2% in Kenya and 13·5% in Uganda. Among facilities that provided ART services, we found average annual OPD volume increases of 7·2% in Kenya and 5·6% in Uganda, with simultaneous annual increases of 13·7% and 12·5% in ART volumes. We did not find a statistically significant relationship between annual changes in OPD services and the presence, growth, overall, or relative size of ART services. However, in a subgroup analysis, we found that Ugandan hospitals that offered ART services had statistically significantly less growth in OPD visits than Ugandan hospitals that did not provide ART services.

**Conclusions:**

Our findings suggest that ART services in Kenya and Uganda did not have a statistically significant deleterious effects on OPD services between 2007 and 2012, although subgroup analyses indicate variation by facility type. Our findings are encouraging, particularly given recent recommendations for universal access to ART, demonstrating that expanding ART services is not inherently linked to declines in other health services in sub-Saharan Africa.

## Background

HIV/AIDS has been a leading cause of death and disability in sub-Saharan Africa since the mid-1990s [1]. However, due to an unprecedented global response, the rate of new HIV infections has declined and an estimated 19·1 million life-years have been saved by interventions such as antiretroviral therapy (ART) [2]. Such gains have been driven by a rapid increase in HIV-specific development assistance for health (DAH), which rose in real terms from $1·4 billion in 2000 to $11 billion in 2015 [3]. Access to and enrollment in ART programs markedly increased in the last decade, [1, 4] providing life-saving treatment for people living with HIV throughout the world. Yet amid this success, considerable debate exists about how this massive scale-up of ART has affected the provision of non-HIV services [5–7]. With HIV-targeted DAH stagnating since 2010 [3] and new treatment guidelines recommending universal access to ART, [8] understanding if and where the scale-up of ART services has had beneficial or detrimental health system effects is critical.

Previous studies provide conflicting evidence on how the scale-up of ART has affected health systems in sub-Saharan Africa. Many argue that the scale-up of ART strengthened overall health system capacity by increasing resource availability at health facilities, improving supply chains and bolstering support for human resources [9–12]. By contrast, other studies have found that scarce resources were diverted away from other health needs and toward largely vertical HIV treatment programs [7, 13, 14]. Most commonly, these findings focused on human resources for health (HRH), [15–17] documenting substantial employee shifts from public facilities to non-governmental organizations (NGOs) and HIV-focused treatment programs due to higher wages, greater prestige, and the opportunity to provide improved medical care [17–20].

Mixed results from existing research suggest there may be a complex interplay between HIV and non-HIV services. For instance, volumes of maternal and reproductive health services such as family planning and antenatal care increased amid rising ART volumes in Zambia, but immunization services experienced fewer gains at facilities providing ART services [21]. Similarly, HIV funding was correlated with gains in maternal services such as prenatal blood testing, but decreases in immunization rates in sub-Saharan Africa [15]. Among rural health centers in Rwanda, the provision of HIV services appeared to have little effect on other patient services, [22] while one study in Uganda noted that facilities with funding from the US President’s Emergency Plan for AIDS Relief (PEPFAR) was associated with an increased number of deliveries [23]. Another recent paper analyzing the effect of PEPFAR investments on non-HIV services at the district-level in Uganda found that high PEPFAR investment was associated with small declines in several non-HIV outputs such as pediatric outpatient visits, TB tests, and inpatient deliveries [24].

In this study, we further examine the interaction between facility-based ART and non-ART outpatient department (OPD) services, filling an important information gap for key stakeholders as they develop policies aimed at reaching universal ART coverage. Drawing from unique, nationally-representative facility datasets in Kenya and Uganda, two countries with significant HIV epidemics, we assess trends in ART and non-ART OPD services at the country level and across facility types from 2007 to 2012. Specifically, we examine the impact of facility-based ART services on the utilization of non-ART OPD services, and investigate how various factors, such as facility location and ownership, influence this relationship.

## Methods

### Facility sample and instrument

Our study used facility-level data collected in Kenya and Uganda as part of the Access, Bottlenecks, Costs and Equity (ABCE) project. For each country, nationally-representative facility samples were constructed using a two-step, stratified random sampling process detailed elsewhere [25, 26]. The sample included publicly- and privately-owned facilities across all levels of care.

Each facility completed the six-part ABCE Facility Survey. This survey included cross-sectional data on facility characteristics, equipment availability, and pharmaceutical stocks. It also requested retrospective longitudinal data (obtained from facility registries) on the number of pre-ART, ART, and OPD visits for the last five fiscal years. Facility data collection took place between April and November 2012 using DatStat Illume Survey Manager 5.1 (DatStat Inc., Seattle WA).

### Definition of key variables

The two key variables used for this analysis were ART visits and OPD visits. For the purposes of this analysis, pre-ART visits were included with ART visits as they were most commonly seen within the ART clinic, utilising the same ART resources and staff. From this point forward, “ART visits” refers to the sum of ART and pre-ART visits, unless otherwise indicated. OPD visits excluded any ART and pre-ART visits, making the ART and OPD visit variables mutually exclusive. We included both adult and child visits for both ART and OPD.

### Analysis

For the purpose of this analysis, we used each country’s health system structure to categorize facilities into hospitals and health centers/primary care facilities; here we refer to the latter facilities as health centers for brevity. The hospital category included sub-district, district, provincial, private hospitals, and nursing homes in Kenya, and district, regional referral, and private hospitals in Uganda. The health center category included health centers, health posts, clinics, and dispensaries in Kenya, and health center IIs, health center IIIs, and health center IVs in Uganda. We excluded national referral hospitals, stand-alone laboratories, pharmacies, and voluntary counseling and testing (VCT) centers given their differences in operational capacities and production processes.

After processing the data to identify and address data entry errors, less than 15% of annual ART and OPD volumes included in this analysis were missing. These values, as well as other key missing variables of interest, were imputed with Amelia II in R, [27] using lags and leads of two years. We ran this model 50 times, and used the median values across these 50 imputations to generate the descriptive statistics presented below. We excluded facilities with fewer than two years of reported data for OPD visits or ART visits from our analysis.

For each facility-year (*t)*, we calculated the annual percent change relative to the previous year ($\frac{visit{s}_t-{ visit s}_{t-1}}{visit{s}_{t-1}}\times 100$) for both ART and OPD visits. We used these results to estimate the average annual percent change in ART and OPD visits across sampled hospitals and health centres in each country. We chose to use a weighted average based on the patient volume in year *t-1* to control for undue influence of smaller facilities.

In our primary analysis, we used a mixed-effects regression model to assess the impact of ART service provision on OPD visits, controlling for facility characteristics. To account for health system differences, we chose to model each country separately. In addition, within each country we performed a subgroup analysis, modeling hospitals and health centers independently, to capture differences in service complexity. Our dependent variable was defined as the annual percent change in OPD visit volume relative to the previous year. For both the primary and subgroup analyses, we ran four different iterations of our model, capturing different aspects of the potential impact of an ART program on non-ART OPD services (Table 1).

**Table 1:** Definition of ART program characteristic variables included in regression analyses

Variable	Definition
ART program presence	Binary indicator reflecting the presence (or absence) of an ART program in a given facility-year.
Overall ART program size	Facility’s total number of pre-ART visits and ART visits (included as the logged number of visits) as an indicator of ART program size for a given facility-year.
Relative ART program size	Facility’s total number of ART visits over number of non-ART OPD visit as an indicator of relative size of the ART program in comparison to the non-ART OPD.
ART growth/rate of scale-up	The annual percent change (year-on-year) in total ART visits to reflect the pace of ART scale-up in a given facility.

We specified the following hierarchical model:

$$ {Y}_{t,j,i}={\beta}_0+{\beta}_1\cdotp AR{T}_{t, ji}+\boldsymbol{C}\cdotp {\boldsymbol{X}}_{\boldsymbol{i},\boldsymbol{j}}+{\beta}_2\cdotp t+{\gamma}_{j,i}+{\delta}_j+{\varepsilon}_{t,j,i} $$

where *Y* is the annual percent change in OPD visits for facility *i* and calendar year *t* (relative to year *t* − 1) in district or county *j*; *ART* is one of the four variables described above (Table 1) to characterize the ART program for facility *f* and calendar year *t*; ***X*** is a vector of covariates including facility-type used in the sampling procedure, facility ownership (public or private/NGO), and location (urban or rural) for facility *i*; *β*_2_is a fixed effect for calendar year *t*; *γ* is a random effect for facility *i*; and *δ* is a random effect for district (Uganda) or county (Kenya) *j*. For facilities that started providing ART during the panel, the year they started providing ART was dropped from the analysis when analyzing percent change, given that percent change from zero is undefined. In the analysis of all facilities using the binary indicator of the presence of an ART program, we include facility and district weights in our analysis. In our specification of the above model, we nested facility-years within facilities and facilities within districts or counties. For all models we used robust standard errors.

All analyses were run in Stata version 13·0 (StataCorp, College Station, Texas) using the MI commands using datasets generated by the Amelia imputation.

## Results

### Sample description

Our final sample included 321 health facilities (140 in Kenya and 181 in Uganda), and a total of 1544 facility-years (666 in Kenya and 878 in Uganda). This included 26 health facilities that opened and 19 facilities that began offering ART services during the study period.

Table 2 provides an overview of health facility characteristics stratified by country, facility type, and the availability of ART services at the time of survey administration. In both Kenya and Uganda, a greater percentage of facilities that provided ART services were publicly owned. Facilities that provided ART services were also substantially larger than those facilities that did not provide ART services, even within the same facility type strata. Not surprisingly, this often translated to additional services, particularly at health centers, including increased laboratory capacity and outreach programs. Across both countries and facility types, health facilities that offered ART programs saw, on average, more patients per day in their OPD clinics than ART clinics.

**Table 2:** Facility characteristics by country and facility type

Indicator	Kenya				Uganda			
	Hospitals		Health centers		Hospitals		Health centers	
	ART	Non-ART	ART	Non-ART	ART	Non-ART	ART	Non-ART
Facility characteristics								
Number of facilities	26	25	25	64	31	22	17	111
Percent urban	81%	96%	52%	38%	81%	91%	29%	21%
Percent publicly owned	77%	20%	84%	50%	55%	9%	88%	70%
Facility physical infrastructure								
Functional electricity connection	100%	96%	96%	84%	100%	95%	76%	53%
Improved water source and sanitation	100%	96%	91%	94%	100%	95%	94%	75%
Facility services								
On-site lab capability	100%	100%	92%	77%	100%	86%	100%	77%
Outreach program	81%	40%	76%	42%	94%	55%	100%	88%
Human resources (Median and IQR)								
Number of medical staff	42	14	10	4	126	11	19	7
	(20-137)	(8-45)	(8-14)	(2-7)	(95-230)	(6-22)	(16-27)	(4-10)
Patient volumes (Median and IQR)								
Non-ART OPD visits per day	69	20	31	12	185	22	52	26
	(35-165)	(7-42)	(16-56)	(5-25)	(90-346)	(13-83)	(38-87)	(15-46)
ART visits per day	13	-	5	-	25	-	2	-
	(2-36)		(1-11)		(4-102)		(<1-6)	
ART + non-ART visits per medical staff per day	1.3	1.0	3.7	3.0	1.8	1.8	3.1	3.8
	(0.8-2.4)	(0.7-1.4)	(2.7-5.4)	(1.5-5.4)	(0.9-2.4)	(1.0-4.3)	(2.5-4.1)	(2.4-6.7)

Values reflect year in which data was collected or the last fiscal year if the variable was collected over the five-year period. Ranges in human resource and patient volumes are reported within parentheses

### Descriptive results

**Fig. 1:**
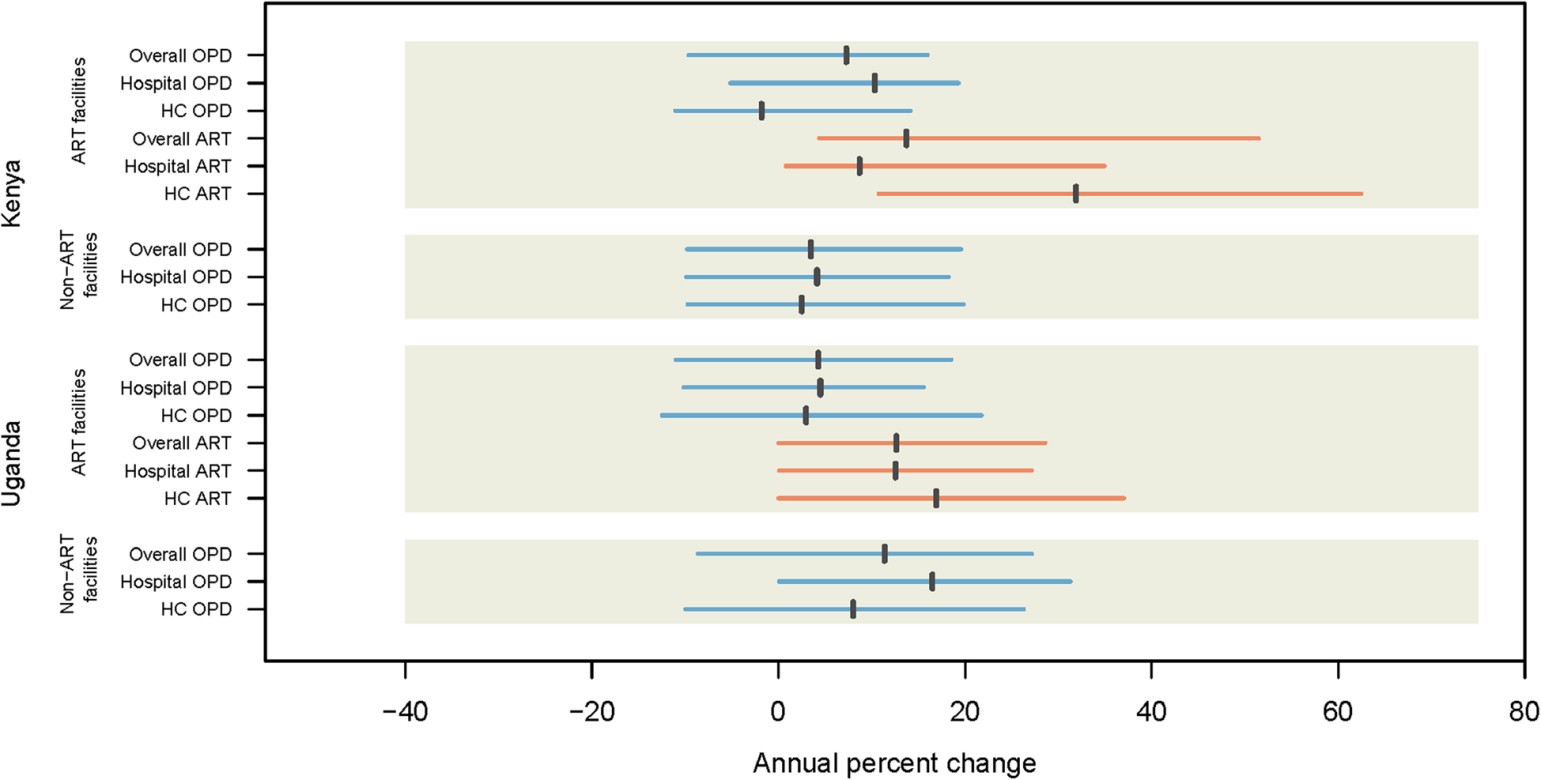
Annual percent change in non-ART OPD and ART visits in Kenya and Uganda. *Vertical black lines* represent weighted average. *Horizontal colored lines* represent interquartile range. Values are not adjusted for any covariates

Between 2007 and 2012, OPD volumes increased at a majority of facilities across Kenya and Uganda (Fig. 1). In Kenya, OPD visits increased at 59% of facilities with ART services and 61% of facilities that did not provide ART. At the same time, we found larger increases in the average annual growth of OPD visits in Kenyan facilities that provided ART services (7·3%) than those that did not (3·5%). In Uganda, 56% of facilities with ART services reported increased OPD growth, while increases were noted at 73% of health facilities that did not offer ART services. Contrary to our findings in Kenya, average annual growth of OPD visits was larger in Ugandan facilities that did not provide ART services compared to those that did (11·4% vs. 4.3%).

Looking more specifically at facility type, we found that in Ugandan hospitals, average annual OPD visits increased more than three times faster in hospitals that did not provide ART services (16·5% vs. 4·5%). Faster growth in OPD visits was also observed among Ugandan health centers that did not provide ART services (8·0% vs. 3·0%)). In Kenya, a very different pattern emerged. Average annual OPD visits increased by 10·3% at hospitals that provided ART services and 4·1% at those that did not. Interestingly, relatively little OPD growth was observed at lower-level facilities in Kenya, irrespective of ART service provision (2·5% average annual increase among non-ART facilities and 1·8% average annual decrease among facilities with ART).

ART visits generally increased at a faster pace than OPD visits. In both Kenya and Uganda, ART visits increased at an average that exceeded a 10% per year. Health centres saw a larger average annual increase in ART visits than hospitals, rising 31·9% and 16·9% per year in Kenya and Uganda, respectively. For hospitals, we found an average annual increase of ART visits of 8·7% in Kenya and 12·5% in Uganda.

### Mixed-effects regression results

In assessing the relationship between ART services and OPD visits at the country level in mixed-effects regression models, we found that neither the presence, rate of growth, overall, nor relative size of ART programs had a statistically significant impact on OPD visits in Kenya or Uganda (Table 3).

**Table 3:** Regression results for the effect of ART program existence and size on the annual rate of change in non-ART OPD visits

ART program measure	Kenya Coefficient (95% confidence interval)			Uganda Coefficient (95% confidence interval)		
	Hospitals	Health Centers	Pooled	Hospitals	Health Centers	Pooled
Presence of ART program	6.14 (−19.69, 31.97)	24.06 (−130.23, 178.36)	6.94 (−56.34, 70.21)	−34.88 (−59.54, −10.22)	42.27 (−255.94, 350.48)	27.93 (−184.78, 240.64)
Percent change in ART visits	0.03 (−0.13, 0.20)	0.03 (−0.18, 0.23)	0.03 (−0.11, 0.16)	0.00 (−0.21, 0.20)	0.17 (−1.73, 2.06)	0.16 (−1.58, 1.91)
Total ART visits (logged)	−5.84 (−30.06, 18.37)	−0.03 (−14.79, 14.73)	−2.78 (−14.62, 9.06)	0.43 (−2.28, 3.13)	0.93 (−12.81, 14.67)	0.17 (−5.04, 5.38)
Relative size of ART program	−14.83 (−152.74, 123.08)	−14.58 (−33.05, 3.88)	−14.09 (−52.64, 24.45)	−2.47 (−5.24, 0.31)	−40.62 (−193.87, 112.63)	−2.51 (−8.12, 3.11)

Each row shows the coefficients and 95% confidence intervals for a different construct of the independent variable measuring ART services. Results control for location (urban or rural) and ownership (public or private/NGO). Pooled results also control for type of facility (hospitals or health centers)

In subgroup analyses based on facility type (hospital and health centers), we did not find a statistically significant relationship between ART services and OPD growth in Kenya (Table 3). This result held true in all four model specifications of ART services (presence, growth, overall, and relative size). In Uganda, subgroup analyses showed that OPD visits were not statistically significantly affected by ART clinic growth and size (overall and relative OPD size). However, while Ugandan health centers did not appear to be impacted by the presence of an ART program, hospitals providing ART services were found to have slower growth in OPD visits compared to those hospitals that did not provide ART service, equating to an OPD growth deficit of 34.88 percentage points (95% confidence interval − 59·54, −10·22; *p* < 0·01) between the two types of hospitals.

## Discussion

To our knowledge, this is the first study to assess the effects of facility-based ART programs on trends in OPD services using nationally-representative facility datasets in sub-Saharan Africa. On average, we observed substantial increases for both ART and OPD volumes between 2007 to 2012 in both Kenya and Uganda. Furthermore, we found that the presence, scale-up, and size (overall and relative to the OPD clinic) of facility-based ART programs had no statistically significant effects on OPD visits over time. Overall, our study suggests that facility-based ART services were introduced and scaled up without having significantly negative effects on OPD volumes in Kenya and Uganda, a result that supports efforts to expand ART services in sub-Saharan Africa.

Sub-group analyses examining health centres and hospitals independently in both Kenya and Uganda generally revealed results consistent with the primary analysis across all health facilities. However, we found that the presence of ART services in Ugandan hospitals appeared to negatively affect OPD growth. The reason for this finding is unclear, but we do note some potentially important differences between hospitals in Uganda that provided ART and those that did not. First, the majority (55%) of hospitals offering ART services were public, while most non-ART hospitals were private (91%). This finding is not a surprise given the way in which ART services were initially rolled out at large public hospitals throughout Uganda and Kenya, before being offered in lower level facilities. However, private institutions may have had differential capacities or desire to expand services given more decentralized decision-making process [28] and different patient-mixes [29]. Second, non-ART hospitals were generally smaller than hospitals providing ART and had small provider to patient ratios (Table 2). It is possible that the smaller facilities simply had more capacity for growth, and were able to expand at a faster rate than the larger institutions. Lastly, it is important to consider that other contextual factors may significantly influence this finding, and the fact that this was noted in only one of the subgroup analyses suggest that further investigation is warranted.

Several past studies support our broader findings, offering a number of explanations as to how ART services expanded without crowding out OPD services. First, many facilities may have had the slack capacity to accommodate more patients prior to the introduction and scale-up of ART, allowing for an influx of additional patients without substantially straining facility resources [30, 31]. Facility managers also may have instituted mechanisms for reallocation of resources within facilities, such as task-shifting among medical staff [5, 32, 33]. Further, the largely vertical funding and implementation of ART programs may have shielded other facility resources from being used for ART [5, 34]. Lastly, HIV-targeted funds may have increased facility supplies and employed additional medical staff specifically for ART services, thus preventing a crowd-out of other types of non-HIV services [9].

Although there has been a large debate on the effect of ART scale-up and vertical programming on the larger health system, the majority of published literature assessing the effects of ART services at the facility level supports our results. Most studies found no significant deleterious effects on the utilization of non-ART services with the introduction of ART services, [22, 35] and some have even shown a positive effect of ART on non-ART services [23]. Much of the research suggesting a negative impact of ART programs on the larger health system focuses on qualitative assessments of health worker migration from the public to private sector, and analyses of how funding allocation changed with the increased focus on HIV/AIDS [8, 16, 18, 20]. However, the impact of ART services may also vary by the service of interest (i.e. inpatient, immunization, antenatal care etc.) [15, 21]. Furthermore, different types of HIV services may have differential effects on non-HIV services, [36] and as we found here, the impact may also vary by facility-type. Finally, we should be wary of a possible non-linear relationship between ART and ODP visits. Currently, many health facilities in Kenya and Uganda are operating with low technical efficiency, indicating some slack capacity that can accommodate ART patients without affecting the volume of other services [30]. Yet both Uganda and Kenya recently updated their guidelines to extend ART eligibility to all people living with HIV, which may substantially increase the strain on the health system and begin affecting other types of services [37, 38].

In an era of stagnating DAH for HIV/AIDS, there is sizeable interest in identifying way for further scaling up HIV services while continuing to provide and expand non-HIV services in a complementary manner [30]. This issue will likely continue to attract attention given the recent update of WHO HIV treatment guidelines, which expand ART eligibility to all people living with HIV [8]. Experiences in Kenya and Zambia show that integrating ART with other health services may be one strategy for supporting continued growth in patient volumes without a corresponding increase in medical staff [39–41]. However, these experiences also show that integration alone cannot overcome existing human resource and infrastructure shortages; instead such integration can lead to greater occupational stress due to increased workload for providers and reduced time with patients [41]. Increased efficiency through improved use of space and staff time, better teamwork and accountability, task-shifting, expanded training, and improved patient satisfaction are all strategies that may support continued ART expansion without negatively impacting existing programs [20, 39–41]. Going forward, it will be important to monitor how new models of care alter the relationship between ART and other services, to both avoid potential strains on resources and maximize synergies.

The findings of this study should be viewed in light of some limitations. First, we could not account for potential shifts in quality of services provided during the introduction and scale-up of ART. The potential exists that in order to meet increased patient loads, less time was spent with patients, and quality of care suffered [16, 41]. On the other hand, prior studies demonstrate that service quality can improve amid investments in laboratories, provider trainings, supply chains, and facility infrastructure, all of which occurred in Kenya and Uganda during their scale-ups of ART [16, 42]. Second, record-keeping and data quality varied substantially by facility, and it is possible that facilities with ART have benefited from investments in improved health information systems [43]. However, in comparing rates of data missingness and entry errors across facilities, we did not find evidence of systematic differences between facilities with and without ART. Finally, we do not truly know what would have happened to OPD services in the absence of ART at facilities that provided both services. Without this counterfactual, we sought to compare facilities with ART to those without ART. However, it is possible that facilities without ART fundamentally differed from facilities with ART. We sought to control for these differences by including several facility characteristics in our analyses, but we could not account for every possible factor, such as task shifting and changes in the catchment population of each facility. Therefore, some unobserved confounding factors may remain.

## Conclusions

Drawing from a nationally-representative facility data set in Kenya and Uganda, we found that the presence, scale-up, overall, and relative size of facility-based ART programs had no statistically significant impact on OPD visits. At the national level, we did not observe a “crowding-out” of OPD services as ART expanded both within facilities and to new facilities. Recent changes in ART recommendations may result in an additional influx of patients seeking ART services, and our study suggests that this may be possible without negatively affecting OPD services. Nonetheless, ongoing monitoring of facility-specific needs and resource use will be critical to both improving service provision and minimising strains to health systems.

## Abbreviations

ABCE: Access, Bottlenecks, Costs and Equity project
ANC: Antenatal care
ART: Antiretroviral therapy
DAH: Development assistance for health
HIV/AIDS: Human immunodeficiency virus/Acquired immune deficiency syndrome
NGO: Non-governmental organization
OPD: Outpatient department
PEPFAR: US President’s Emergency Plan for AIDS Relief
PMTCT: Prevention of mother-to-child transmission of HIV
VCT: Voluntary counseling and testing
WHO: World Health Organization

## References

[1] Wang H, Wolock T, Carter A, Nguyen G, Hmwe HK, Gakidou E (2016). Estimates of global, regional, and national incidence, prevalence, and mortality of HIV, 1980–2015: the Global Burden of Disease Study 2015. Lancet HIV.

[2] Murray CJL, Ortblad KF, Guinovart C, Lim SS, Wolock TM, Roberts DA (2014). Global, regional, and national incidence and mortality for HIV, tuberculosis, and malaria during 1990–2013: a systematic analysis for the Global Burden of Disease Study 2013. Lancet.

[3] Dieleman JL, Schneider MT, Haakenstad A, Singh L, Sadat N, Birger M (2016). Development assistance for health: past trends, associations, and the future of international financial flows for health. Lancet.

[4] UNAIDS (2016). Global AIDS Update 2016.

[5] Yu D, Souteyrand Y, Banda MA, Kaufman J, Perriëns JH (2008). Investment in HIV/AIDS programs: Does it help strengthen health systems in developing countries?. Glob Health.

[6] Levine R, Oomman N (2009). Global HIV/AIDS funding and health systems: searching for the win-win. J Acquir Immune Defic Syndr.

[7] Shiffman J, Berlan D, Hafner T (2009). Has aid for AIDS raised all health funding boats?. J Acquir Immune Defic Syndr.

[8] World Health Organization (WHO). Guideline on when to start antiretroviral therapy and on pre-exposure prohylaxis for HIV. Geneva: WHO; 2015. Available from: http://apps.who.int/iris/bitstream/10665/186275/1/9789241509565_eng.pdf 26598776

[9] Druce N, Nolan A (2007). Seizing the big missed opportunity: linking HIV and maternity care services in sub-Saharan Africa. Reprod Health Matters.

[10] Samb B, Celletti F, Holloway J, Van Damme W, De Cock KM, Dybul M (2007). Rapid expansion of the health workforce in response to the HIV epidemic. N Engl J Med.

[11] Justman JE, Koblavi-Deme S, Tanuri A, Goldberg A, Gonzalez LF, Gwynn CR (2009). Developing laboratory systems and infrastructure for HIV scale-up: a tool for health systems strengthening in resource-limited settings. J Acquir Immune Defic Syndr.

[12] Piot P, Kazatchkine M, Dybul M, Lob-Levyt J (2009). AIDS: lessons learnt and myths dispelled. Lancet.

[13] Shiffman J (2008). Has donor prioritization of HIV/AIDS displaced aid for other health issues?. Health Policy Plan.

[14] England R (2007). Are we spending too much on HIV?. BMJ.

[15] Grépin KA (2012). HIV donor funding has both boosted and curbed the delivery of different non-HIV health services in sub-Saharan Africa. Health Aff.

[16] Brugha R, Kadzandira J, Simbaya J, Dicker P, Mwapasa V, Walsh A (2010). Health workforce responses to global health initiatives funding: a comparison of Malawi and Zambia. Hum Resour Health.

[17] Hanefeld J, Musheke M (2009). What impact do Global Health Initiatives have on human resources for antiretroviral treatment roll-out? A qualitative policy analysis of implementation processes in Zambia. Hum Resour Health.

[18] World Health Organization Maximizing Positive Synergies Collaborative Group. An assessment of interactions between global health initiatives and country health systems. Lancet 2009; 373:2137-69. 10.1016/S0140-6736(09)60919-3 19541040

[19] Chima CC, Franzini L (2016). Spillover effect of HIV-specific foreign aid on immunization services in Nigeria. Int Health.

[20] Assefa Y, Jerene D, Lulseged S, Ooms G, Damme WV (2009). Rapid scale-up of antiretroviral treatment in Ethiopia: successes and system-wide effects. PLoS Med.

[21] Brugha R, Simbaya J, Walsh A, Dicker P, Ndubani P (2010). How HIV/AIDS scale-up has impacted on non- HIV priority services in Zambia. BMC Public Health.

[22] Shepard DS, Zeng W, Amico P, Rwiyereka AK, Avila-Figueroa C (2012). A controlled study of funding for human immunodeficiency virus/acquired immunodeficiency syndrome as resource capacity building in the health system in Rwanda. Am J Trop Med Hyg.

[23] Kruk ME, Jakubowski A, Rabkin M, Elul B, Friedman M, El-Sadr W (2012). PEPFAR programs linked to more deliveries in health facilities by African women who are not infected with HIV. Health Aff.

[24] Luboga SA, Stover B, Lim TW, Makumbi F, Kiwanuka N, Lubega F (2016). Did PEPFAR investments result in health system strengthening?.

[25] Institute for Health Metrics and Evaluation (IHME). Health Service Provision in Uganda: Assessing Facility Capacity, Costs of Care, and Patient Perspectives. Seattle, WA; 2014. Available from: http://www.healthdata.org/policy-report/health-service-provision-uganda-assessing-facility-capacity-costs-care-and-patient

[26] Institute for Health Metrics and Evaluation (IHME). Health Service Provision in Kenya: Assessing Facility Capacity, Costs of Care, and Patient Perspectives. Seattle, WA; 2014. Available from: http://www.healthdata.org/policy-report/health-service-provision-kenya-assessing-facility-capacity-costs-care-and-patient

[27] Amelia II: A Program for Missing Data | Honaker | Journal of Statistical Software. [cited 2016 Aug 8]. Available from: https://www.jstatsoft.org/article/view/v045i07

[28] Leonard KL, Masatu MC, Vialou A (2007). Getting doctors to do their best: the roles of ability and motivation in health care quality. J Hum Resour.

[29] Konde-Lule J, Gitta SN, Lindfors A, Okuonzi S, Onama VO, Forsberg BC (2010). Private and public health care in rural areas of Uganda. BMC Int Health Hum Rights.

[30] Di Giorgio L, Moses MW, Fullman N, Wollum A, Conner RO, Achan J (2016). The potential to expand antiretroviral therapy by improving health facility efficiency: evidence from Kenya, Uganda, and Zambia. BMC Med.

[31] Kioko U (2013). Efficiency in Kenya: Implications for Fiscal Space.

[32] World Health Organization (2007). Task shifting to tackle health worker shortages.

[33] Callaghan M, Ford N, Schneider H (2010). A systematic review of task- shifting for HIV treatment and care in Africa. Hum Resour Health.

[34] Schneider H, Coetzee D, Van Rensburg D, Gilson L (2010). Differences in antiretroviral scale up in three South African provinces: the role of implementation management. BMC Health Serv Res.

[35] Matsubayashi T, Manabe YC, Etonu A, Kyegombe N, Muganzi A, Coutinho A (2011). The effects of an HIV project on HIV and non-HIV services at local government clinics in urban Kampala. BMC Int Health Hum Rights.

[36] Wilson N (2015). Can disease-specific funding harm health? In the shadow of HIV/AIDS service expansion. Demography.

[37] Ministry of Health, National AIDS & STI Control Programme. Guidelines on use of Antiretroviral Drugs for Treating and Preventing HIV Infection in Kenya 2016. Kenya: NASCOP; 2016.

[38] Republic of Uganda Ministry of Health (MOH). Consolidated Guidelines for Prevention and Treatment of HIV in Uganda. Kampala: MOH; 2016.

[39] Topp SM, Chipukuma JM, Chiko MM, Matongo E, Bolton-Moore C, Reid SE (2013). Integrating HIV treatment with primary care outpatient services: opportunities and challenges from a scaled-up model in Zambia. Health Policy Plan.

[40] Odeny TA, Penner J, Lewis-Kulzer J, Leslie HH, Shade SB, Adero W (2013). Integration of HIV care with primary health care services: effect on patient satisfaction and stigma in Rural Kenya, integration of HIV care with primary health care services: effect on patient satisfaction and stigma in Rural Kenya. AIDS Res Treat.

[41] Mutemwa R, Mayhew S, Colombini M, Busza J, Kivunaga J, Ndwiga C (2013). Experiences of health care providers with integrated HIV and reproductive health services in Kenya: a qualitative study. BMC Health Serv Res.

[42] Okero FA, Aceng E, Madraa E, Serutoke J (2003). Scaling up antiretroviral therapy: Experience in Uganda.

[43] Oomman N, Bernstein M, Rosenzweig S. Following the funding for HIV/AIDS: A Comparative analysis of the funding practices of PEPFAR and teh Global Fund and World Bank MAP in Mozambique, Uganda and Zambia. Washington, DC: Center for Global Development; 2007.

